# Current status of medical oncology in Japan and changes over the most recent 7-year period: results of a questionnaire sent to designated cancer care hospitals

**DOI:** 10.1093/jjco/hyab135

**Published:** 2021-08-20

**Authors:** Makoto Arai, Izumi Ohno, Koji Takahashi, Meng Meng Fan, Akinobu Tawada, Chikashi Ishioka, Yuichi Takiguchi

**Affiliations:** Department of Medical Oncology, Graduate School of Medicine, Chiba University, Chiba, Japan; Department of Gastroenterology, Tokyo Women's Medical University Yachiyo Medical Center, Chiba, Japan; Department of Medical Oncology, Graduate School of Medicine, Chiba University, Chiba, Japan; Department of Medical Oncology, Graduate School of Medicine, Chiba University, Chiba, Japan; Department of Medical Oncology, Graduate School of Medicine, Chiba University, Chiba, Japan; Department of Medical Oncology, Graduate School of Medicine, Chiba University, Chiba, Japan; Chiba Prefectural University of Health Sciences, Chiba, Japan; Department of Clinical Oncology, Tohoku University Graduate School of Medicine, Sendai, Japan; Department of Medical Oncology, Graduate School of Medicine, Chiba University, Chiba, Japan

**Keywords:** medical oncology, chemotherapy, molecular-targeted therapy, immune-checkpoint inhibitors

## Abstract

**Background:**

According to a questionnaire sent to Designated Cancer Care Hospitals in Japan in 2013, only 39.4% of the institutes had medical oncology departments. Furthermore, most of these medical oncology departments were primarily responsible for the treatment of limited disease categories and the administration of newly developed therapeutic modalities, including molecular-targeted therapy. The aim of the present study was to update these previous findings and to clarify the changes over the intervening 7-year period.

**Methods:**

The questionnaire was sent to all 393 Designated Cancer Care Hospitals on 13 March 2020. Similar to the previous questionnaires, questions were asked regarding the presence of a medical oncology department, the number of physicians in the department and the degrees of responsibility for drug therapies provided by medical oncologists to adult patients with solid cancers.

**Results:**

In total, 270 institutions (68.7%) responded. Overall, 145 of these 270 institutions (53.7%) had medical oncology departments, representing a significant increase compared with the results of the previous study (*P* < 0.01). Among the institutions with a medical oncology department, these departments were responsible for the administration of over 30% of all cytotoxic and molecular-targeted drug therapies for extragonadal germ cell tumors, cancers of unknown primary site, soft tissues, head and neck, esophagus, stomach, colon and rectum, and pancreas as well as the administration of immune checkpoint inhibitors (ICI) for microsatellite instability-high tumors, cancers of the stomach, esophagus and head and neck, and melanoma.

**Conclusion:**

The proportion of institutes with medical oncology departments in Japan has increased. In addition, the responsibility of medical oncology departments has expanded to include newly emerging drugs, such as ICIs.

## Teaser

The proportion of institutes with medical oncology departments in Japan has increased significantly and their responsibility has expanded to include newly emerging drugs.

## Introduction

As the number of patients with cancer continues to increase in Japan (1), requests for knowledge and treatments for cancer are dramatically expanding. This increase in demand is partly due to the increase in the number of complex patients, for example those with an advanced age (2), those receiving polypharmacy (3,4) and those with multiple primary cancers (5), and partly because of the drastic increase in the availability of new drugs for cancer treatment (6). At present, cytotoxic drugs, molecular-targeted drugs and immune checkpoint inhibitors (ICI) are available, and complex combination therapies using these drugs are being performed for the treatment of various cancers. In addition, the progress of human genomics using next-generation sequencing has allowed an increase in precision medicine for specific genome alterations (7), leading to highly specialized cancer treatments. Based on information obtained from cancer genome studies, tumor-agnostic treatments for patients with solid tumors with microsatellite instability (MSI) or neurotrophic tyrosine kinase receptors fusions are now recommended. Therefore, current medical oncology requires non-organ-specific treatment strategies.

Medical oncologists are defined as specialists in the diagnosis of cancer and its treatment using drug therapies (8). New biological therapies and molecular-targeted therapies are now being developed at incredible speeds; therefore, the roles of medical oncologists are expanding. The Japanese Society of Medical Oncology (JSMO) was founded in 2002 and began certifying medical oncologists in 2006, resulting in the certification of 1455 medical oncologists throughout Japan as of April 2020. Considering the fact that the number of JSMO certificated medical oncologists in 2013 was 867, the number of medical oncologists in Japan has increased drastically. In the Basic Plan to Promote Cancer Control Programs published by the Ministry of Health, Labor and Welfare in 2018, two of the primary objectives are the promotion of radiation therapy/chemotherapy and the training of doctors specialized in this area. We sent a questionnaire to 393 Designated Cancer Care Hospitals, which have been designated by the Ministry of Health, Labor and Welfare (Japan) as playing major roles in providing specialized cancer treatment, establishing a system for cooperation with regional cancer treatments and providing information on cancer treatment to patients and inhabitants. To meet the above-mentioned objectives, the Basic Plan to Promote Cancer Control Programs by the Ministry of Health, Labor and Welfare (Japan) calls for the employment of JSMO-certified medical oncologists, who are capable of treating patients with any type of cancer, in these Designated Cancer Care Hospitals (9).

In 2013, we investigated the status of medical oncology in Japan by sending a questionnaire to Designated Cancer Care Hospitals (10). Among the respondents, 39.4% of the institutions had medical oncology departments containing a median of three physicians. JSMO-certified medical oncologists were employed in 156 of the 267 institutions (58.4%), with a median of one in the department (ranging from 0 to 24). Although most of the medical oncology departments were primarily responsible for the treatment of limited disease categories, the responsibility for administering molecular-targeted drugs was relatively high. The aim of the present study was to clarify the change in the status of medical oncology in Japan over the intervening 7-year period between 2013 and 2020.

## Patients and methods

The questionnaire, along with a cover letter describing the purpose of the present study, was sent to all Designated Cancer Care Hospitals. Although the number of Designated Cancer Care Hospitals was 397 in the previous study conducted in 2013, the certification of 32 hospitals was revoked and the certification of 28 hospitals was newly approved during the 7-year period; thus, the questionnaire was sent to 393 hospitals. The questionnaire was addressed to ‘the principal person in charge of cancer practice’ at each institution. The first mailing was on 13 March 2020, with returns obtained from 177 institutions as of 8 May 2020, at which time the second mailing was performed for the remaining institutions. Finally, 270 institutions (68.7%) had returned their answers as of 8 June 2020. This response was almost the same as that of the previous study (68.0%).

The questionnaire was almost the same as the one used in the previous study. Questions were asked regarding the following items: (i) the presence of a Department of Medical Oncology in the hospital; (ii) the number of staff-physicians in the department, if present; (iii) the presence of JSMO-certified medical oncologists in the institute, along with the number of such specialists, and (iv) the primarily, secondarily and tertiarily responsible departments, in terms of patient number, for the use of chemotherapy, molecular-targeted therapy, ICI therapy, hormonal therapy and cytokine therapy for each of 22 specific cancers. Compared with the previous questionnaire, we added ‘ICI therapy’ for cancers of the head and neck, esophagus, stomach, lung, breast, and kidney and melanoma, and ‘MSI-high tumor’ was added as a new category. In addition, we changed ‘bevacizumab therapy’ to ‘molecular-targeted therapy’ for brain and ovarian tumors. The Department of Medical Oncology was defined as an internal medical department responsible for the cross-sectional management of non-organ-specific cancers. In the questionnaire, only data exclusively limited to adult solid cancers were requested. We set the department and modality according to each tumor-organ. For example, in lung cancer, we set that the departments which performed chemotherapy were medical oncology, thoracic surgery, respiratory medicine and others and that the modalities for treatment of lung cancer were cytotoxic drug, ICI and molecular-targeted drugs.

**Figure 1:**
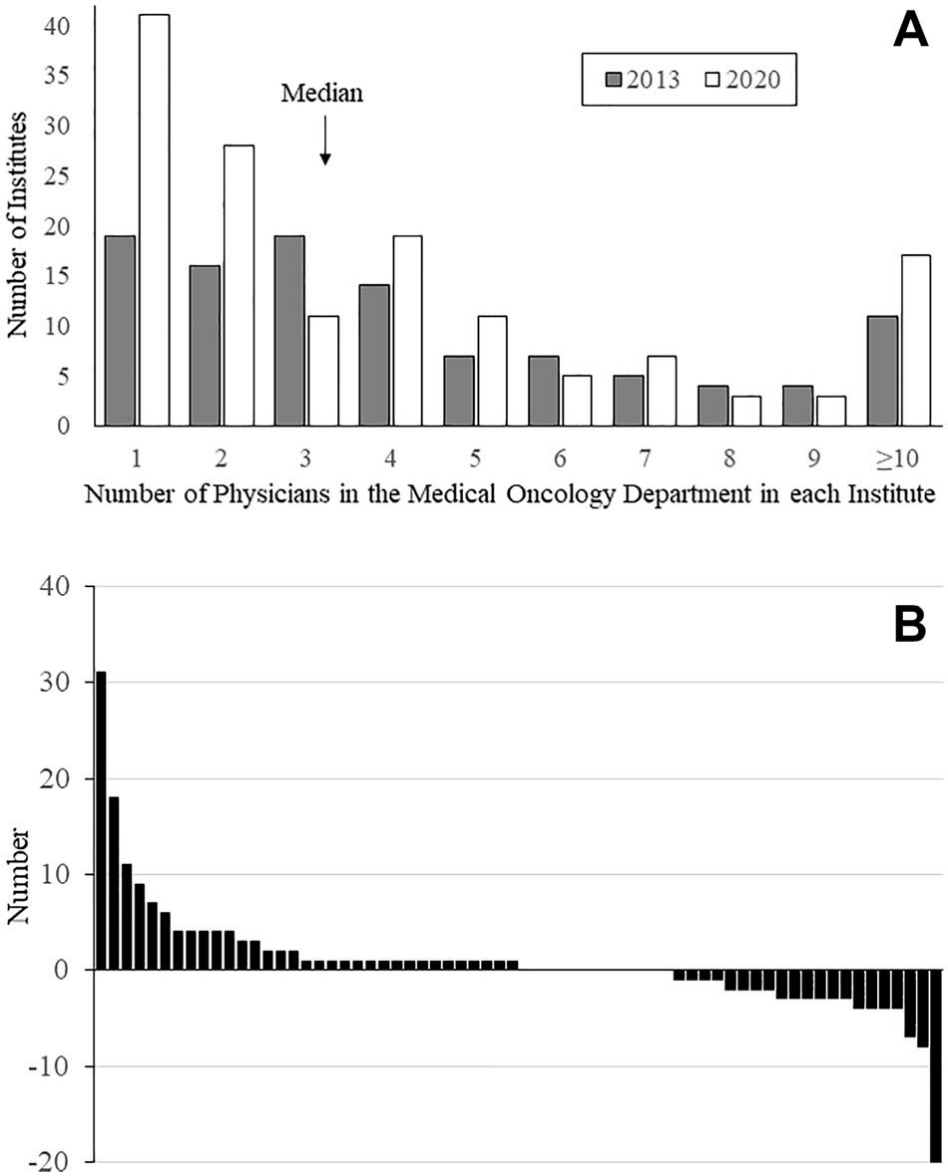
(A) Distribution of the number of physicians working in medical oncology departments. The shaded and open bars represent the numbers in 2013 and 2020, respectively. (B) Change in the number of physicians during the 7-year period in 66 institutes where medical oncology departments had already been established in 2013.

First, we compared the percentage of hospitals with a medical oncology department to that in the previous study using the Student *t*-test, and we checked the distribution of the number of physicians in medical oncology departments in Designated Cancer Care Hospitals in Japan as well as the relationship between the percentages of institutions with medical oncology departments according to the number of JSMO-certified medical oncologists. We also analyzed the proportion of institutions in which a given department of the institution was primarily, secondarily and tertiarily responsible, in terms of patient number, for the use of a given therapeutic modality for each disease. We then compared these values to those obtained in the 2013 questionnaire using the Student *t*-test; differences with a *P* value <0.05 (two-tailed) were judged as being significant using SPSS Statistics 26 (IBM, Armonk, NY, USA).

## Results

### Changes in the presence of medical oncology departments and JSMO-certified medical oncologists

**Figure 2:**
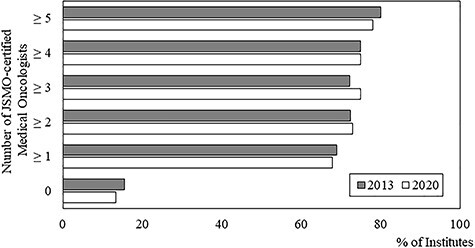
Percentages of institutes with medical oncology departments according to the number of JSMO-certified medical oncologists. The shaded and open bars represent the numbers in 2013 and 2020, respectively.

Overall, 145 of the 270 institutions (53.7%) had medical oncology departments, with a median of three physicians. This percentage was significantly higher than that (107/270, 39.3%) of the previous study (*P* < 0.01). The number of staff members employed in the medical oncology departments increased from 525 in 107 institutes to 747 in 145 institutes during the 7-year intervening period. The distribution of the number of physicians working in the medical oncology department is shown in Fig. 1A. The median number of physicians in the medical oncology departments was three. In 69 of the 145 medical oncology departments (47.6%), only one or two physicians were employed. Sixty-six of the institutes’ medical oncology departments had already been established in 2003. The change in the number of staff members at these 66 institutes is shown in Fig. 1B and Supplementary Table S2. The sum of staff members at these 66 institutes increased from 363 to 413 during the 7-year intervening period, and the median number of physicians in these departments was 4; among these institutes, 34 medical oncology departments (50.7%) had four or more staff members. The number of physicians per medical oncology department therefore seems to be stable, with only mild fluctuations at most institutes. However, some departments experienced drastic changes (either increases or decreases) in the numbers of physicians employed by the medical oncology department. In 192 of the 265 institutions (72.5%, five institutions did not answer this specific question), JSMO-certified medical oncologists were employed, with a median of 2 certified medical oncologists working in the 265 institutes (ranging from 0 to 39). Compared with the previous study, the percentage of institutions with JSMO-certified medical oncologists was significantly higher than that of the previous study (72.5 vs. 58.4%; *P* < 0.01, chi-square test). When limited to institutions with medical oncology departments, 92 of the 107 institutions (86.0%) had employed JSMO-certified medical oncologists, with a median number of 2 (ranging from 0 to 24). The proportions of institutions with medical oncology departments according to the number of JSMO-certified medical oncologists are shown in Fig. 2. In the institutes without JSMO-certified medical oncologists, the proportion of institutions with medical oncology departments was lower (13.3%) than those with JSMO-certified medical oncologists (*P* < 0.01, chi-squared test), similar to the situation in 2013.

### Responsibilities of medical oncology departments for cancer drug therapy

**Figure 3:**
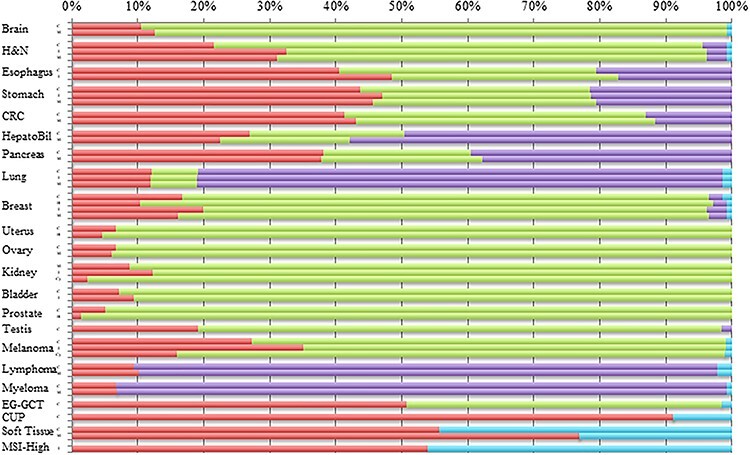
The primary responsibility of a given department in an institute for the treatment of a specific disease among the institutes with a medical oncology department. The red, green, purple and sky-blue bars represent medical oncology departments and sub-departments of surgery specific to the corresponding organ system, sub-departments of internal medicine specific to the corresponding organ system and others, respectively (for the specific department names, see Supplementary Table S1). In head and neck cancer, ‘purple’ represents the Department of Oral Surgery, not Internal Medicine. C, M, I, H and Cy represent cytotoxic chemotherapy, molecular-targeted therapy, ICIs, hormonal therapy and cytokine therapy, respectively. H&N, head and neck cancer; CRC, colorectal cancer; HepatoBil, hepato-biliary cancer; EG-GCT, extragonadal germ cell tumors; CUP, cancers of unknown primary site; MSI-H, MSI-high cancer. In Supplementary Fig. S3, those in all institutes were shown.

The responsibilities, in terms of patient number, of a given department for providing a given therapy for a specific disease are summarized in Supplementary Table S1. In this table, the percentage of institutions in which a given department of the institution was primarily, secondarily or tertiarily responsible for providing a given therapy for a specific disease is presented in each column, with comparisons to the situations in all the institutions that responded to the questionnaire as well as all the institutions with a medical oncology department. To clarify the responsibility of medical oncology departments, the primary departments responsible for administering cytotoxic and molecular-targeted chemotherapy are shown in Fig. 3 for institutions with a medical oncology department and in Supplementary Fig. S3 for all the institutions. In Fig. 3, which contains data for all the institutions with a medical oncology department, the medical oncology department was responsible for over 30% of the cytotoxic and molecular-targeted drugs administered for the treatment of cancers of the head and neck, esophagus, stomach, colon, rectum and pancreas and of ICIs administered for the treatment of esophageal cancer and melanoma. In Supplementary Fig. S3, which shows the data for all the institutions, the medical oncology departments were primarily responsible for over 30% of the treatments for extragonadal germ cell tumors (EGT), cancers of unknown primary site (CUP), soft tissue sarcoma, and MSI-high tumors.

**Figure 4:**
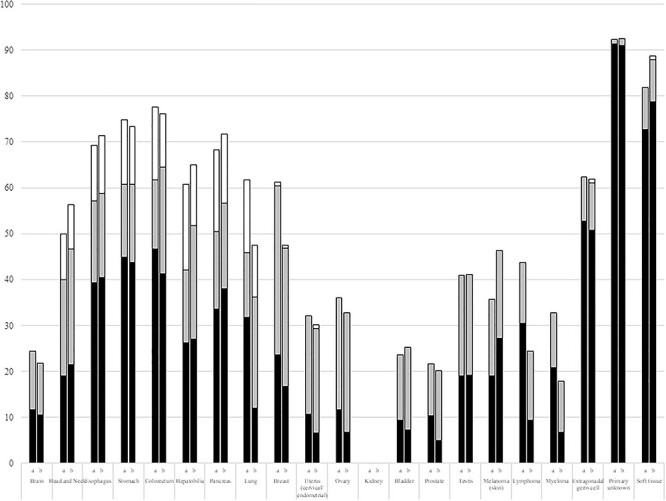
Comparison of the responsibilities of medical oncology departments in institutes with medical oncology departments between 2013 and 2020 in cytotoxic drugs and (Supplementary Fig. S4) molecular-targeted drugs. The closed, shaded and open bars represent primary, secondary and tertiary responsibility, respectively. ‘a’ and ‘b’ show the data in 2013 and 2020, respectively.

In the institutes with medical oncology departments, compared with the results obtained seven years ago, the sums of primary, secondary, and tertiary responsibility for cytotoxic drug therapy provided by medical oncologists are shown in Fig. 4. In Supplementary Fig. S4, those for molecular-targeted drugs were shown. The responsibility of medical oncology departments for the treatment of patients with head and neck tumors tended to be higher than it was seven years ago (*p* = 0.08, chi-squared test). In contrast, the responsibilities of medical oncology departments for administering cytotoxic and molecular-targeted drug therapies for patients with lung cancer, breast cancer, malignant lymphoma, or multiple myeloma were significantly lower (*p* < 0.05).

### Contributions of medical oncology departments to the use of ICIs

ICIs have been used for cancers of the head and neck, esophagus, stomach, lung, breast, kidney, and urinary tract; melanoma; and MSI-high tumors in Japan at March in 2020. The primary departments responsible for administering ICI treatments are shown in Table 1. For MSI-high tumors, stomach and esophageal cancer, medical oncology departments were responsible for over 40% of ICI treatments. In contrast, medical oncology departments were responsible for less than 30% of ICI treatments for lung, breast, kidney, and bladder cancer. Compared to the responsibility of medical oncology department in cytotoxic chemotherapy, those in ICI were higher in head and neck, esophagus, stomach, lung, kidney, urinary tract, and melanoma, although there was no significant difference. The responsibility of medical oncology department for ICI treatment in breast cancer was same as that for cytotoxic chemotherapy.

**Table 1:** Responsibility of medical oncology departments in terms of patient number for the administration of ICIs

Disease	Number of Institutes	Order of responsibility, in terms of patient number (%)				
		Total		Primary	Secondary	Tertiary
MSI-high tumors	128	94 (73.4)		69 (53.9)	25 (19.5)	0 (0)
Stomach	136	99 (72.8)		64 (47.1)	18 (13.2)	17 (12.5)
Esophagus	128	91 (71.1)		62 (48.4)	15 (11.7)	14 (10.9)
Head and neck	132	80 (60.6)		42 (32.6)	27 (20.5)	10 (7.5)
Melanoma (skin)	114	63 (55.3)		40 (35.1)	23 (20.2)	0 (0)
Lung	142	65 (45.8)		17 (12.0)	35 (24.6)	13 (9.2)
Breast	130	52 (40.0)		26 (20.0)	26 (20.0)	0 (0)
Kidney	138	40 (29.0)		17 (12.3)	23 (16.7)	0 (0)
Bladder	138	35 (25.4)		13 (9.4)	22 (20.2)	0 (0)

## Discussion

The present study disclosed the current situation of medical oncology departments in Designated Cancer Care Hospitals in Japan as of 2020. As a similar study was previously conducted in 2013, a comparison of the two studies enabled us to uncover the changes that occurred during the intervening 7-year period. As new drugs for chemotherapy have been developed recently, the therapy for cancer has changed to be complicated. We speculated that the necessity of the experts for cancer treatment would increase in association with the complexity of chemotherapy. To reveal it, we have tried to perform the change of the status of medical oncology for the first time in seven years. The percentage of questionnaire respondents in 2020 was reasonably high at 68.7% (270/393) and was comparable to that obtained in 2013 (68.0%, 270/397). Among Designated Cancer Care Hospitals, 51 institutes (49 regional centers and 2 national cancer institutes) are expected to play a central role. The response rate of 51 institutes was 76.5%, which was not different from that of other institutes (68.5%). Next, to reveal the regional difference, we divided Japan to 10 areas (Hokkaido, Tohoku, Kousinetsu, Kanto, Tokai, Hokuriku, Kinki, Chugoku, Kyushu/Okinawa areas) and compared the response rates in each region. The response rate in Hokuriku area was highest (93.8%, 15 out of 16), in contrast, that in Chugoku area was lowest (60.6%, 20 out of 33), which showed the significant difference (p = 0.016, Pearson's chi-square test), although there was no significant difference between Chugoku area and other area. Overall, 53.7% (145/270) of the institutes had medical oncology departments, representing a significant increase compared with the results of the previous study (39.4%). In addition, the total number of physicians who worked in the medical oncology departments increased from 525 to 747. However, the number of physicians per medical oncology department remained unchanged during the 7-year period, with a median value of 3 in both 2013 and 2020 and mean values of 4.9 and 5.2 in 2013 and 2020, respectively. This situation seems to be similar to that reported in South Korea, where 47.7% (32/68) of surveyed institutions had medical oncology departments with a mean number of physicians per department of 4 (ranging from 1 to 21) (11). In some medical oncology departments, the number of physicians per department changed drastically, either increasing or decreasing, during the 7-year period. We failed to add an item to the questionnaire to query the reason for the change in physician number, as we failed to anticipate such a situation when designing the questionnaire. Institutional reorganization, for example, the division of an oncology/hematology department into independent departments or vice versa, may explain this situation. Overall, the number of JSMO-certified medical oncologists per institution increased from a median of 1 (ranging from 0 to 24) to 2 (ranging from 0 to 39), suggesting that the numerical increases in medical oncology departments and medical oncologists might have been accompanied by an increased quality of care.

Similar to the results obtained in 2013, the present study conducted in 2020 also demonstrated that medical oncology departments are primarily responsible for the treatment of cancers of unknown primary site and soft-tissue sarcomas and secondarily responsible for the treatment of EGTs. In addition, in the 2020 survey, medical oncology departments were also secondarily responsible for the treatment of MSI-high tumors. In 2013, they were significantly more responsible for molecular-targeted therapy than for chemotherapy for head and neck cancer, suggesting that medical oncologists were responsible for the administration of newly developed therapies (10). Moreover, the present study demonstrated a greater responsibility for administering ICI treatment, compared with chemotherapy, for head/neck, esophageal, breast, and urothelial cancers and melanoma. These results are concordant with the results from the 2013 study, which suggested that medical oncology departments were specifically responsible for newly developed therapies. In contrast to the increased responsibility of medical oncology departments for the treatment of head/neck cancer, their responsibility for the treatment for lung cancer, breast cancer, malignant lymphoma and multiple myeloma decreased during the course of the 7-year intervening period, possibly because of changes in the roles of medical oncology departments. As these departments are especially required for the administration of new modality therapies, the responsibility for administering already established treatment modalities might accordingly return to organ-specific departments.

The presence of JSMO-certified medical oncologists was related to the presence of a medical oncology department. A position paper by the European Society for Medical Oncology clarified that quality cancer care should be provided by a multidisciplinary team (MDT) of highly qualified healthcare professionals and that medical oncologists are core members of MDTs (12). The presence of JSMO-certified medical oncologists might lead to an improved quality of cancer care provided by MDTs, in addition to providing opportunities for research, prevention, diagnosis and supportive care; these qualities might lead some institutes to establish medical oncology departments.

The present study had some limitations. The study conducted in 2020 and its comparisons with the results obtained in 2013 only reflect the quantifiable statuses of medical oncology departments. Although it would be difficult to evaluate the quality of medical oncology departments directly because of the lack of an established methodology, the evaluation of medical oncology departments by other healthcare professionals employed by the same institute might partly answer this question. Further investigation is needed to clarify the quality of cancer treatment provided by medical oncology departments.

In conclusion, compared with 7 years ago, the proportion of institutes with medical oncology departments in Japan has increased significantly, and the total number of physicians employed in medical oncology departments has increased. The number of JSMO-certificated medical oncologists who were employed in Designated Cancer Care Hospitals has also increased. As previously suggested by the study performed in 2013, the primary contribution of medical oncology departments in 2020 was the administration of newly developed therapeutic modalities and expanded treatment indications for ICIs. Further development of both the quantity and quality of medical oncology is necessary to improve cancer care in Japan further.

## Supplementary Material

20210418MAMOTableS1_hyab135

SupplementaryTableS2_hyab135

FigS3_hyab135

FigS4_hyab135

AraiSUPPL1_hyab135

## Abbreviations

JSMO, Japanese Society of Medical Oncology; ICI, immune checkpoint inhibitor; irAE, immune-related adverse events.
