# Supplementary material for: Current status of medical oncology in Japan and changes over the most recent 7-year period: results of a questionnaire sent to designated cancer care hospitals
Source: Jpn J Clin Oncol. 2021 Aug 20;51(11):1622–7. doi: 10.1093/jjco/hyab135 (PMC8558914; doi:10.1093/jjco/hyab135)
Supplement: SupplementaryTableS2_hyab135 [file supplementarytables2_hyab135.doc]

**Supplementary Table S2: The Numbers of Physicians in the Medical Oncology Departments in 2013 and 2020**

| Numbers of Physicians | | Change in Numbers of Physicians  from 2013 to 2020 |
| --- | --- | --- |
| 2013 | 2020 |
| 35 | 66 | 31 |
| 3 | 21 | 18 |
| 26 | 37 | 11 |
| 1 | 10 | 9 |
| 9 | 16 | 7 |
| 8 | 14 | 6 |
| 5 | 9 | 4 |
| 4 | 8 | 4 |
| 3 | 7 | 4 |
| 2 | 6 | 4 |
| 3 | 7 | 4 |
| 3 | 6 | 3 |
| 9 | 12 | 3 |
| 3 | 5 | 2 |
| 7 | 9 | 2 |
| 1 | 3 | 2 |
| 4 | 5 | 1 |
| 4 | 5 | 1 |
| 2 | 3 | 1 |
| 3 | 4 | 1 |
| 4 | 5 | 1 |
| 1 | 2 | 1 |
| 2 | 3 | 1 |
| 2 | 3 | 1 |
| 3 | 4 | 1 |
| 3 | 4 | 1 |
| 4 | 5 | 1 |
| 2 | 3 | 1 |
| 3 | 4 | 1 |
| 1 | 2 | 1 |
| 3 | 4 | 1 |
| 3 | 4 | 1 |
| 1 | 2 | 1 |
| 4 | 4 | 0 |
| 5 | 5 | 0 |
| 10 | 10 | 0 |
| 1 | 1 | 0 |
| 2 | 2 | 0 |
| 1 | 1 | 0 |
| 2 | 2 | 0 |
| 5 | 5 | 0 |
| 8 | 8 | 0 |
| 2 | 2 | 0 |
| 3 | 3 | 0 |
| 1 | 1 | 0 |
| 2 | 1 | -1 |
| 2 | 1 | -1 |
| 8 | 7 | -1 |
| 5 | 4 | -1 |
| 3 | 1 | -2 |
| 6 | 4 | -2 |
| 7 | 5 | -2 |
| 4 | 2 | -2 |
| 4 | 1 | -3 |
| 4 | 1 | -3 |
| 4 | 1 | -3 |
| 5 | 2 | -3 |
| 12 | 9 | -3 |
| 4 | 1 | -3 |
| 22 | 18 | -4 |
| 5 | 1 | -4 |
| 9 | 5 | -4 |
| 5 | 1 | -4 |
| 11 | 4 | -7 |
| 9 | 1 | -8 |
| 21 | 1 | -20 |
