# Supplementary figures and images for: Current status of medical oncology in Japan and changes over the most recent 7-year period: results of a questionnaire sent to designated cancer care hospitals
Source: Jpn J Clin Oncol. 2021 Aug 20;51(11):1622–7. doi: 10.1093/jjco/hyab135 (PMC8558914; doi:10.1093/jjco/hyab135)

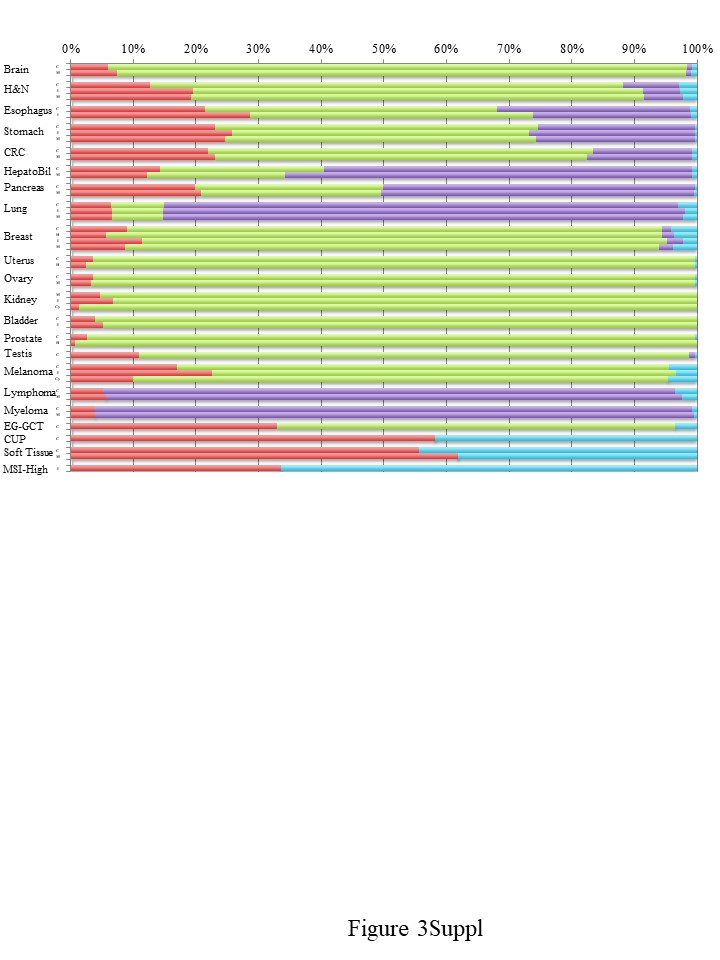

Supplement: FigS3_hyab135 [file figs3_hyab135.jpeg]

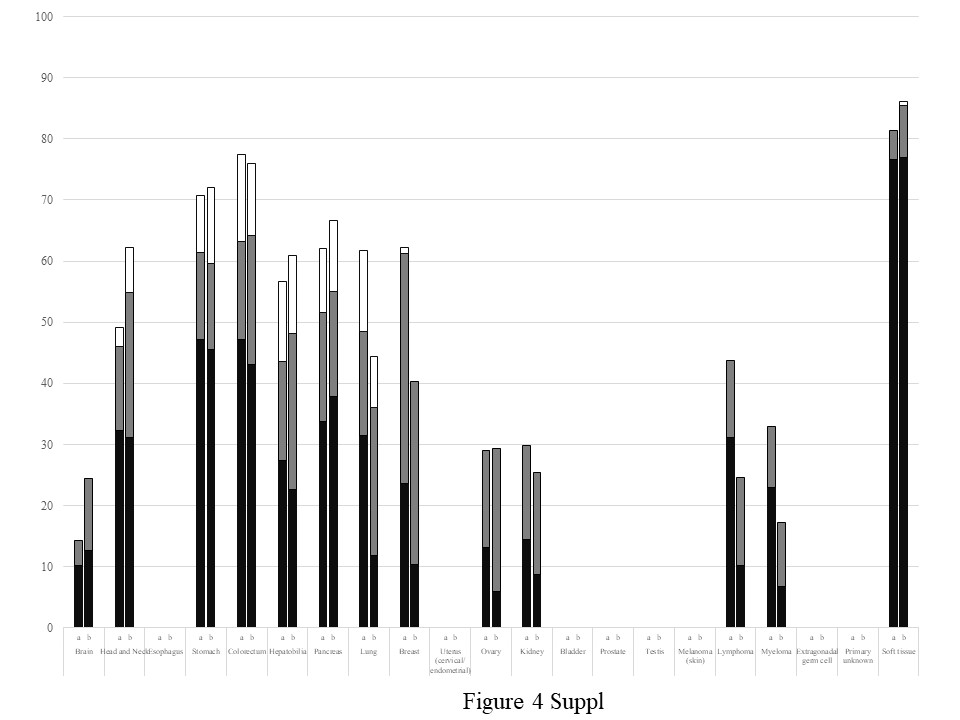

Supplement: FigS4_hyab135 [file figs4_hyab135.jpeg]
