# Supplementary material for: Current status of medical oncology in Japan and changes over the most recent 7-year period: results of a questionnaire sent to designated cancer care hospitals
Source: Jpn J Clin Oncol. 2021 Aug 20;51(11):1622–7. doi: 10.1093/jjco/hyab135 (PMC8558914; doi:10.1093/jjco/hyab135)
Supplement: AraiSUPPL1_hyab135 [file araisuppl1_hyab135.doc]

Supplementary File1:

The names of the institutes and, in parentheses, the respondents who provided consent are acknowledged here as follows: Fukushima Medical University (Shigehira Saji), Aomori Prefectural Central Hospital (Masaki Munakata), Akita University Hospital (Kazuhiro Shimazu), Yamagata Prefectural Central Hospital (Yuriko Sasahara), Miyagi Cancer Center (Yasuko Murakawa), Tohoku University Hospital (Chikashi Ishioka), Iwate Medical University Hospital (Hiroaki Itamochi), Tochigi Cancer Center (Seigo Yukisawa), Gunma University Hospital (Norifumi Tsukamoto), The Cancer Institute Hospital Of JFCR (Shunji Takahashi), Kanagawa Cancer Center (Rika Sakai), Niigata Cancer Center Hospital (Satoru Nakagawa), Toyama Prefectural Central Hospital, Kanazawa University Hospital (Seiji Yano), Fukui Prefectural Hospital (Yasukazu Kawai), Yamanashi Prefectural Central Hospital (Masaki Iino), Shinshu University Hospital (Tomonobu Koizumi), Gifu University Hospital, Shizuoka Cancer Center (Hirofumi Kanemura), Aichi Cancer Center Hospital (Kei Muro), Mie University Hospital (Toshiro Mizuno), Shiga General Hospital (Kosuke Asagoe), Kyoto Prefectural University of Medicine (Takeshi Ishikawa), Kyoto University Hospital (Shigemi Matsumoto), Osaka International Cancer Institute, Hyogo Cancer Center (Koji Matsumoto), Nara Medical University Hospital (Masatoshi Kanno), Wakayama Medical University Hospital (Hiroki Ueda), Tottori University Hospital (Hisashi Suyama), Hiroshima University Hospital (Kazuhiko Sugiyama), Yamaguchi University Hospital (Shigefumi Yoshino), Kagawa University Hospital, National Hospital Organization Kyushu Cancer Center (Taito Esaki), Kyusyu University Hospital (Eiji Baba), Nagasaki University Hospital, Kumamoto University Hospital (Kisato Nosaka), Oita University Hospital (Shuichi Hironaka), University of Miyazaki Hospital (Ayumu Hosokawa), Kagoshima University Hospital (Shinichi Ueno), Hakodate Municipal Hospital, Sapporo City General Hospital (Michio Nakamura), Sunagawa City Medical Center, Asahikawa-Kosei General Hospital (Kazuya Sato), Japanese Red Cross Kitami Hospital (Minoru Kamibayashi), Obihiro-Kosei Hospital (Ryusuke Matsumoto), Kushiro City General Hospital (Tomohiro Sakashita), Hakodate Goryokaku Hospital (Akinori Takagane), KKR Sapporo Medical Center (Susumu Sogabe, Tetsuya Kojima), Sapporo Medical University Hospital (Junji Kato), Teine Keijinkai Hospital (Atsushi Ishiguro), Hokkaido University Hospital (Hirotoshi Akita), Asahikawa City Hospital, Kushiro Rosai Hospital (Kazuaki Harada), Hirosaki University Hospital (Kazuhiro Hosoi), Towada City Hospital (Michinaga Takahashi), Iwate Prefectural Central Hospital (Satoshi Kato), Iwate Prefectural Miyako Hospital, Oohunato Hospital (Toru Hoshida), Iwate Prefectural Kuji Hospital (Chihiro Tono), Sendai Medical Center (Takao Suzuki), Osaki Citizen Hospital (Yasuhiro Sakamoto), Japanese Red Cross Ishinomaki Hospital (Hisatsugu Ohmori), Odate Municipal General Hospital (Hitoshi Ogasawara), Yamagata University Hospital (Takashi Yoshioka), Okitama Public General Hospital (Koichiro Ozawa), Nihonkai General Hospital, Southern Tohoku General Hospital (Yoshinao Takano), Takeda General Hospital, Fukushima Rosai Hospital, Hitachi General Hospital (Masakazu Tsutsumi), University of Tsukuba Hospital (Ikuo Sekine), Tokyo Medical University Ibaraki Medical Center (Nobuyuki Koyama), Jichi Medical University Hospital (Hironori Yamaguchi), Dokkyo Medical University Hospital, Maebashi Red Cross Hospital (Atsushi Takise), Takasaki General Medical Center (Atsushi Naganuma), Shibukawa Medical Center (Toru Yokota), Kiryu Kosei General Hospital, Gunma Prefectural Cancer Center（Kazuhiro Araki), Dokkyo Medical University Saitama Medical Center (Manabu Arai), Saitama Red Cross Hospital, Saitama City Hospital (Katutoshi Sekine), Saitama Medical Center (Hideyuki Ishida), Saitama Hospital (Yumi Nozaki), Saitama Medical University International Medical Center (Yoshihiko Segawa), Fukaya Red Cross Hospital (Hiroshi Ito), Saiseikai Kawaguchi General Hospital (Masanori Matsuda), Chiba University Hospital (Makoto Arai), Chiba Medical Center (Yasuyoshi Toyoda), Funabashi Municipal Medical Center (Satoshi Hirano), Juntendo University Urayasu Hospital (Hidekazu Takahashi), Matsudo City General Hospital (Takashi Saotome), Kimitsu Chuo Hospital (Norio Ureshino), The University of Tokyo Hospital (Kiyoshi Miyakawa), Nippon Medical School Hospital (Kaoru Kubota), St. Luke’s International Hospital, NTT Medical Center Tokyo (Keita Uchida), Nihon University Itabashi Hospital (Katsuhiro Miura), Teikyo University Hospital (Nobuhiko Seki), Ome Municipal General Hospital (Takashi Kumagai), Tokyo Medical University Hachioji Medical Center (Takuya Aoki), Kyorin University Hospital, Juntendo University Hospital, Showa University Hospital (Yutaro Kubota), Keio University Hospital (Yasuo Hamamoto), Tokyo Medical University Hospital (Akinobu Yoshimura), Tokyo Metoropolitan Tama Medical Hospital (Mikio Takamori), The Jikei University Hospital (Singo Yano), Toranomon Hospital (Yuji Miura), Yokohama Rosai Hospital (Hitoshi Arioka), Municipal Citizen’s Hospital (Hiroaki Okamoto), Yokohama City University Hospital (Yasushi Ichikawa), Yokosuka Kyosai Hospital, Fujisawa City Hospital (Nobuko Kusano), Kitasato University Hospital (Jiichiro Sasaki), Odawara Municipal Hospital (Koyama Takashi), Showa University Northern Yokohama Hospital (Hiroo Ishida), Yokohama City Minato Red Cross Hospital, Niigata City General Hospital (Kazuhiko Ito), Niigata University Medical and Dental Hospital (Yasuo Saijyo), Nagaoka Chuo General Hospital, Nagaoka Red Cross Hospital (Kazuhiro Sato), Niigata Prefectural Central Hospital (Akihito Momoi), Kurobe City Hospital, Toyama University Hospital (Ryuji Hayashi), Kouseiren Takaoka Hospital (Kazuhiko Shibata), Takaoka City Hospital (Wataru Fukushima), Tonami General Hospital, Kanazawa Medical Center (Hayato Sugiura), Ishikawa Prefectural Central Hospital (Kunihiro Tsuji), Kanazawa Medical University Hospital (Yoshiharu Motoo), Komatsu Municipal Hospital (Yutaka Matano), University of Fukui Hospital (Eiju Negoro), Fukui Red Cross Hospital (Kanae Nakanishi), Fukui-Ken Saiseikai Hospital (Shun Nakayama), University of Yamanashi Hospital, Fujiyoshida Municipal Medical Center, Saku Central Hospital (Michitaka Nagase), Aizawa Hospital (Masato Nakamura), Nagano Red Cross Hospital (Naoaki Ichikawa), Ina Central Hospital (Nobumichi Takeuchi), Gifu Prefectural General Medical Center (Katsuyuki Kunieda), Ogaki Municipal Hospital (Keitaro Kamei), Gifu Prefectural Tajimi Hospital, Juntendo University Shizuoka Hospital (Kenichiro Tanaka), Shizuoka General Hospital (Keisei Taku), Fujieda Municipal General Hospital (Shigeki Ohata), Seirei Hamamatsu General Hospital (Satoru Nakayama), Hamamatsu University Hospital (Masato Karayama), Iwata City Hospital (Tadasu Tobita), Nagoya Medical Center (Keiji Sugiyama), Nagoya University Hospital (Tomoya Shimokata), Nagoya City University Hospital, Japanese Red Cross Nagoya Daiichi Hospital (Yasuyuki Nakano), Kainan Hospital (Setsuo Utsunomiya), Tosei General Hospital (Kajiguchi Tomohiro), Komaki City Hospital, Toyota Kosei Hospital, Anjo Kosei Hospital (Yuichiro Inagaki), Japanese Red Cross Ise Hospital, Suzuka General Hospital (Keiki Kawakami), Nagahama City Hospital (Yasutaka Takubo), Hikone Municipal Hospital (Satoru Takeji), Shiga University of Medical Science Hospital (Yataro Daigo), Kyoto City Hospital (Toshihiko Kirishima), Japanese Red Cross Kyoto Daini Hospital (Naoki Kakihara), Toyonaka Municipal Hospital (Tomono Kawase), Higashiosaka City Medical Center (Kiyotsugu Iede), Osaka Minami Medical Center（Fumihiko Nakanishi), Kishiwada City Hospital (Tomohiro Ozaki), Osaka City General Hospital (Haruko Daga), Osaka Red Cross Hospital (Takehiko Tsumura), Osaka City University Hospital, Osaka Medical College Hospital (Masahiro Goto), Kindai University Hospital（Kazuhiko Nakagawa), Kansai Medical University Hospital (Takayasu Kurata), Osaka National Hospital (Takeshi Kato), Kobe City Medical Center General Hospital (Hisateru Yasui), Kansai Rosai Hospital (Takashi Ota), The Hospital of Hyogo College of Medicine (Takashi Kijima), Kinki Central Hospital, Nishiwaki Municipal Hospital (Sachiko Kimura), Japanese Red Cross Society Himeji Hospital (Toshihiko Matsumoto), Tenri Hospital, Kindai University Nara Hospital (Takao Tamura), Tottori Prefecture Chuo Hospital, Tottori Prefectural Kousei Hospital (Naoya Noguchi), Yonago Medical Center (Hirokazu touge), Matsue City Hospital (Kenichi Takeda), Shimane Prefectural Central Hospital (Koushi Kawakami), Japanese Red Cross Okayama Hospital (Akihiro Bessho), Kurashiki Central Hospital (Shinichi Nishina), Kawasaki Medical School Hospital (Yoshiyuki Yamaguchi), Hiroshima Prefectural Hospital (Katsunori Shinozaki), Hiroshima Red Cross Hospital & Atomic-Bomb Survivors Hospital, Onomichi General Hospital (Tomoko Suzuki), Fukuyama City Hospital (Hisayuki Endo), Hiroshima City Asa Hospital (Souichi Kitaguchi), Shuto General Hospital, Tokuyama Central Hospital, Yamaguchi Grand Medical Center (Ryuichiro Sudo), Tokushima Prefectural Central Hospital, Tokushima Red Cross Hospital (Hisashi Ishikura), Kagawa Prefectural Central Hospital, Takamatsu Red Cross Hospital, Kagawa Rousai Hospital (Hidetaka Takimoto), Sumitomo Besshi Hospital (Minoru Matubara), Saiseikai Imabari Hospital, Ehime University Hospital (Yoshihiro Yakushijin), Ehime Prefectural Central Hospital, Kochi Health Sciences Center (Yuji Negoro), Hata Kenmin Hospital, Kyushu Medical Center (Hozumi Shimokawa), Saiseikai Fukuoka General Hospital (Yasunori Emi), Kurume University Hospital (Koji Nagafuji), Omuta City Hospital, Kitakyushu Municipal Medical Center, Hospital of The University of Occupational and Environmental Health, Ureshino Medical Center (Kazutoshi Komiya), Japanese Red Cross Nagasaki Genbaku Hospital (Masaaki Fukuda), Sasebo City General Hospital (Hitoshi Soda), Nagasaki Prefectural Shimabara Hospital (Akitoshi Kinoshita), Kumamoto Medical Center (Kenji Sakai), Saiseikai Kumamoto Hospital (Tatsuma Morikita), Kumamoto Rosai Hospital (Hirotaka Maruyama), Beppu Medical Center, Oita Red Cross Hospital (Kengo Fukuzawa), Saiseikai Hita Hospital (Satomi Aihara), Nakatsu Municipal Hospital (Yasuo Fukuyama), Miyazaki Prefectural Miyazaki Hospital (Syuji Arita), Miyakonojo Medical Center, Kagoshima Medical Center (Kimiharu Uozumi), Saiseikai Sendai Hospital (Kuniaki Aridome), Kagoshima City Hospital (Junichi Nakazawa), Okinawa Chubu Hospital (Yoshitaka Asakura), Naha City Hospital, National Cancer Center Hospital (Yuichiro Oe), Iwaki City Medical Center, Jichi Medical University Saitama Medical Center (Koichi Suzuki), Todachuo General Hospital, Tokyo Medical And Dental University Medical Hospital (Toshiaki Ishikawa), National Disaster Medical Center, Yokohama City University Medical Center (Satoshi Komiyama), Nagoya City West Medical Center, Aichi Medical University Hospital (Hideyuki Mishima), Handa City Hospital (Yoshito Okada), Uji Tokushukai Hospital (Shuji Yanagida), Osaka General Medical Center (Junji Uchida), Sakai City Medical Center (Junya Fujita), Itami City Hospital (Hitoshi Yoshida), Kobe city Nishi-Kobe Medical Center, Tobata Kyoritsu Hospital (Yuji Maruyama), Sagara hospital (Tetsuhiko Taira).
